# Fowne’s Chemistry for Students

**Published:** 1854-02

**Authors:** 


For sale by Keen & Bro's. J.

---

*Fowne's Chemistry for Students*, edited by Robt. Bridges, M. D. etc Philadelphia: Blanchard & Lea, 1853.

THIS work has been adopted as a text book by the Professor of Chemistry in Rush Med. College, which perhaps is a sufficient recommendation. The London edition was brought out under the supervision of H. Bence Jones, whose name is a guaranty for its accuracy.

We unhesitatingly recommend it to medical students.

